# Efficacy and safety of transarterial chemoembolization plus antiangiogenic- targeted therapy and immune checkpoint inhibitors for unresectable hepatocellular carcinoma with portal vein tumor thrombus in the real world

**DOI:** 10.3389/fonc.2022.954203

**Published:** 2022-11-25

**Authors:** Jin-Kai Feng, Zong-Han Liu, Zhi-Gang Fu, Zong-Tao Chai, Ju-Xian Sun, Kang Wang, Yu-Qiang Cheng, Hong-Fei Zhu, Yan-Jun Xiang, Li-Ping Zhou, Jie Shi, Wei-Xing Guo, Jian Zhai, Shu-Qun Cheng

**Affiliations:** ^1^ Department of Hepatic Surgery VI, Eastern Hepatobiliary Surgery Hospital, Second Military Medical University, Shanghai, China; ^2^ Department II of Interventional Radiology, Eastern Hepatobiliary Surgery Hospital, Second Military Medical University, Shanghai, China; ^3^ Department of Hepatobiliary Surgery, The First Affiliated Hospital, Wenzhou Medical University, Zhejiang, China

**Keywords:** hepatocellular carcinoma (HCC), portal vein tumor thrombus (PVTT), transarterial chemoembolization (TACE), anti-angiogenic targeted therapy, PD-1 inhibitor, immune checkpoint inhibitor (ICI), combination therapy

## Abstract

**Purpose:**

This study aimed to assess the efficacy and safety of a triple therapy that comprises transarterial chemoembolization (TACE), antiangiogenic-targeted therapy, and programmed death-1 (PD-1) inhibitors in a real-world cohort of patients with unresectable hepatocellular carcinoma (HCC) with portal vein tumor thrombus (PVTT).

**Methods:**

Consecutive patients treated with TACE combined with antiangiogenic therapy and PD-1 inhibitors at the Eastern Hepatobiliary Surgery Hospital between June 2019 and May 2021 were enrolled. The baseline characteristics and treatment course of the patients were recorded. The tumor response was evaluated based on the Response Evaluation Criteria in Solid Tumors (RECIST) 1.1 and HCC-specific modified RECIST (mRECIST). The overall survival (OS) and progression-free survival (PFS) of the patients were analyzed using the Kaplan–Meier method. Adverse events (AEs) were assessed according to the National Cancer Institute Common Terminology Criteria for Adverse Events version 5.0.

**Results:**

As of the data cutoff on 30 August 2021, the median follow-up time was 10.0 (3.9–28.4) months. A total of 39 eligible patients were included. The objective response rate (ORR) and the disease control rate (DCR) were 35.9% and 74.4% according to the RECIST 1.1, and 48.7% and 84.6% according to mRECIST criteria, respectively. The median OS and PFS were 14.0 and 9.2 months, respectively. Moreover, 34 (87.2%) patients experienced at least one treatment-related AE and 8 (20.5%) patients experienced grade 3/4 treatment-related AEs. The most common treatment- and laboratory-related AEs were hypertension (46.2%) and decreased albumin (53.8%), respectively. No treatment-related mortality occurred during the study period.

**Conclusions:**

TACE combined with antiangiogenic-targeted therapy and immune checkpoint inhibitors may have promising anticancer activity in unresectable HCC patients with PVTT. AEs were manageable, with no unexpected overlapping toxicities.

## Introduction

Hepatocellular carcinoma (HCC) is the most common primary liver cancer and the fourth-leading cause of cancer-related death worldwide (1). Portal vein tumor thrombus (PVTT) is common in advanced HCC, with a reported incidence of 44%–62.2% (2). If left untreated, the median survival time of these patients is only from 2.7 to 4.0 months (3). PVTT is recognized as an independent prognostic factor for HCC patients (4, 5).

Transarterial chemoembolization (TACE) is the most widely used treatment option for unresectable HCC and has been globally adopted as the standard of care for patients with Barcelona Clinic Liver Cancer (BCLC) stage B HCC (6). Moreover, TACE is proven to be a safe and effective treatment modality for patients with BCLC stage C HCC in the clinical setting (7–9). An established theory holds that TACE stimulates the expression of angiogenic growth factors and promotes the release of an abundance of tumor antigens, which contribute to tumor growth or progression. Since TACE is generally a palliative therapy, it acts as a backbone for the addition of effective systemic therapies aimed at improving survival outcomes.

Recently, increasing studies have investigated the potential clinical role of locoregional–systemic treatments in unresectable HCC patients. Several studies demonstrated that TACE plus sorafenib or lenvatinib significantly improved survival outcomes compared with TACE monotherapy in patients with unresectable HCC (10–12). A single-arm study suggested that the triple therapy approach consisted of PD-1/PD-L1 inhibitors plus radiotherapy, and antiangiogenic therapy appears to be safe with no unexpected adverse events (AEs) (13). A retrospective comparative study found that hepatic arterial infusion chemotherapy (HAIC) combined with PD-1 inhibitors and lenvatinib was associated with a remarkably better treatment response and survival outcomes for patients with advanced HCC compared to patients who received PD-1 inhibitors plus lenvatinib (14). Therefore, the combination of locoregional and systemic therapies is producing exponentially increasing interest in the research field of advanced HCC.

In our study, we focused on a real-world cohort of unresectable HCC patients with PVTT who received a triple therapy approach that comprises TACE plus antiangiogenic-targeted treatment and anti-PD-1 inhibitors (TTP treatment). The therapeutic efficacy and safety of this triple therapy approach were evaluated. We present the following article in accordance with the STROBE reporting checklist.

## Materials and methods

### Study design and patients

This was a single-arm study to evaluate the efficacy and tolerability of a combination treatment of TACE plus antiangiogenic therapy and anti-PD-1 antibodies in unresectable HCC patients associated with PVTT in the real world. Patients who received TTP treatment to treat HCC with PVTT at the Eastern Hepatobiliary Surgery Hospital of Second Military Medical University between June 2019 and May 2021 were reviewed. The study was conducted in accordance with the Declaration of Helsinki (as revised in 2013). The study was approved by the institutional ethics committee of Eastern Hepatobiliary Surgery Hospital, and individual consent for this retrospective analysis was waived. All laboratory serum examination data were collected 3 days before the initial treatment. Imaging evaluation comprised contrast-enhanced computed tomography (CT) or magnetic resonance imaging (MRI) examination within 1 week before the initial treatment. All the data were censored on 30 August 2021.

### Inclusion and exclusion criteria

The inclusion criteria included the following: (I) unresectable HCC and PVTT confirmed radiologically or histologically according to the AASLD practice guidelines (15); (II) Child–Pugh class A or B liver function; (III) Eastern Cooperative Oncology Group (ECOG) performance status 0–1; (IV) at least one measurable tumor lesion as defined by modified Response Evaluation Criteria in Solid Tumors (mRECIST); (V) at least one follow-up imaging assessment; and (VI) at least one cycle of anti-PD-1 antibody treatment. The exclusion criteria included the following: (I) patients who had the triple therapy as an adjuvant treatment after surgery; (II) patients who had initially unresectable disease were downstaged for surgical resection after TTP treatment; (III) history of other malignancies; and (VI) medical and follow-up data were incomplete or unavailable.

### Treatment protocols

TACE was performed according to previously described procedures. Briefly, a 4–5 French catheter was selectively introduced through a femoral artery into the hepatic artery using the Seldinger technique. Arterioportogram was performed to assess tumor staining and vascularity. The tip of the microcatheter was directly advanced into the tumor-feeding arteries depending on the tumor size and location. An emulsion of pirarubicin (30 mg), fluorouracil, oxaliplatin, and lipiodol (10–30 ml; 1–2 ml/cm diameter of the tumor; Lipiodol Ultrafluide, Guerbet, Aulnay-Sous-Bois, France) was infused. Oxaliplatin (100mg or 65 mg/m^2^ body surface area) was dissolved in 5 ml of normal saline and infused slowly with a rate of 1 ml/min. Fluorouracil (500 mg or 330 mg/m^2^ body surface area) was injected slowly within 10 min. Gelfoam fragments were then injected to embolize the tumor-feeding vessels until stasis of blood flow was achieved. The dosages of lipiodol were determined by the body surface area and underlying liver function. On-demand TACE was conducted repeatedly when the intrahepatic lesion was not fully necrotic and the active area was greater than 50% of the baseline until unTACEable progression occurred. UnTACEable progression was defined as the circumstances in which patients were not capable to benefit from TACE, such as Child–Pugh class C, intrahepatic progression, with new lesions not defined as tumor progression.

Antiangiogenic-targeted agents, including tyrosine kinase inhibitors (sorafenib, lenvatinib, and anlotinib) and vascular endothelial growth factor (VEGF) blockade (apatinib), were administered orally. Sorafenib was given 400 mg/day initially and increased to 800 mg/day in a stepwise manner if tolerated. The dosage of lenvatinib was 8 mg/day (<60 kg) or 12 mg/day (≥60 kg) depending on body weight. Anlotinib was prescribed 12 mg/day during weeks 1–2 of each 3-week cycle. Apatinib was prescribed 500 mg/day initially and increased to 750 mg/day if tolerated.

In this study, four types of PD-1 inhibitors (sintilimab, toripalimab, camrelizumab, and tislelizumab) were used at the standard dose intravenously. The first use of PD-1 inhibitors was within 7 days of the initiation of antiangiogenic-targeted drugs. Patients received targeted drugs and PD-1 inhibitors within 3 days before or after the start of TACE. Dosage reduction or the discontinuation of treatment depended on disease progression, unacceptable toxicity, a patient’s withdrawal of consent, or the changes of a treatment plan.

For patients with progressive disease (PD) after TTP therapy, patients could transfer to other recommended combination treatments, such as radiotherapy plus molecular-targeted drugs and PD-1 inhibitor triple therapy, or TACE plus radiotherapy, molecular-targeted drugs and PD-1 inhibitor quadruple therapy, or atezolizumab plus bevacizumab (T+A) first-line therapy. The subsequent therapeutic methods for PD patients were determined after the full discussions of the multidisciplinary treatment meetings, and the final treatment decisions mainly depended on the economic capability of patients.

### Treatment efficacy and safety evaluation

The radiological response was evaluated by dynamic CT or magnetic resonance imaging (MRI) at baseline and every 6–12 weeks after the initial treatment. The tumor response including the objective response rate (ORR) and disease control rate (DCR) was assessed in accordance with the Response Evaluation Criteria in Solid Tumors (RECIST) 1.1 and HCC-specific modified RECIST (mRECIST). The ORR was calculated as the sum of the complete response (CR) and partial response (PR). DCR was defined as the sum of the CR, PR, and stable disease (SD). Overall survival (OS) was defined as the time interval from the date of treatment initiation to the date of death or the most recent follow-up visit. Progression-free survival (PFS) referred to the time interval from treatment initiation to the first radiologically confirmed PD or death. Treatment safety was continuously evaluated by clinical vital signs and laboratory tests. AEs were assessed according to the National Cancer Institute Common Terminology Criteria for Adverse Events version 5.0.

### Statistical analysis

Continuous variables were reported as median (interquartile range) or mean ± standard deviation (SD) and compared using Student’s t-test or the Mann–Whitney U test according to the normality of data. Categorical variables were presented as frequency (percentage) and compared using Pearson’s chi-square test or Fisher’s exact test as appropriate. OS and PFS were estimated using the Kaplan–Meier method. Differences in survival curves were analyzed with a log-rank test. Univariate Cox regression analysis was used to evaluate the significance of potential variables associated with OS and PFS. Variables that were significantly related to OS and PFS (*P* < 0.05) were incorporated into multivariate Cox regression analysis. A two-tailed *P* value < 0.05 was considered statistically significant. All statistical analyses were performed using SPSS 26.0 software (SPSS Inc., Chicago, IL, USA) and GraphPad Prism, Version 8.2.0 (GraphPad, Inc.).

## Results

### Identification and characteristics of study patients

**Figure 1:**
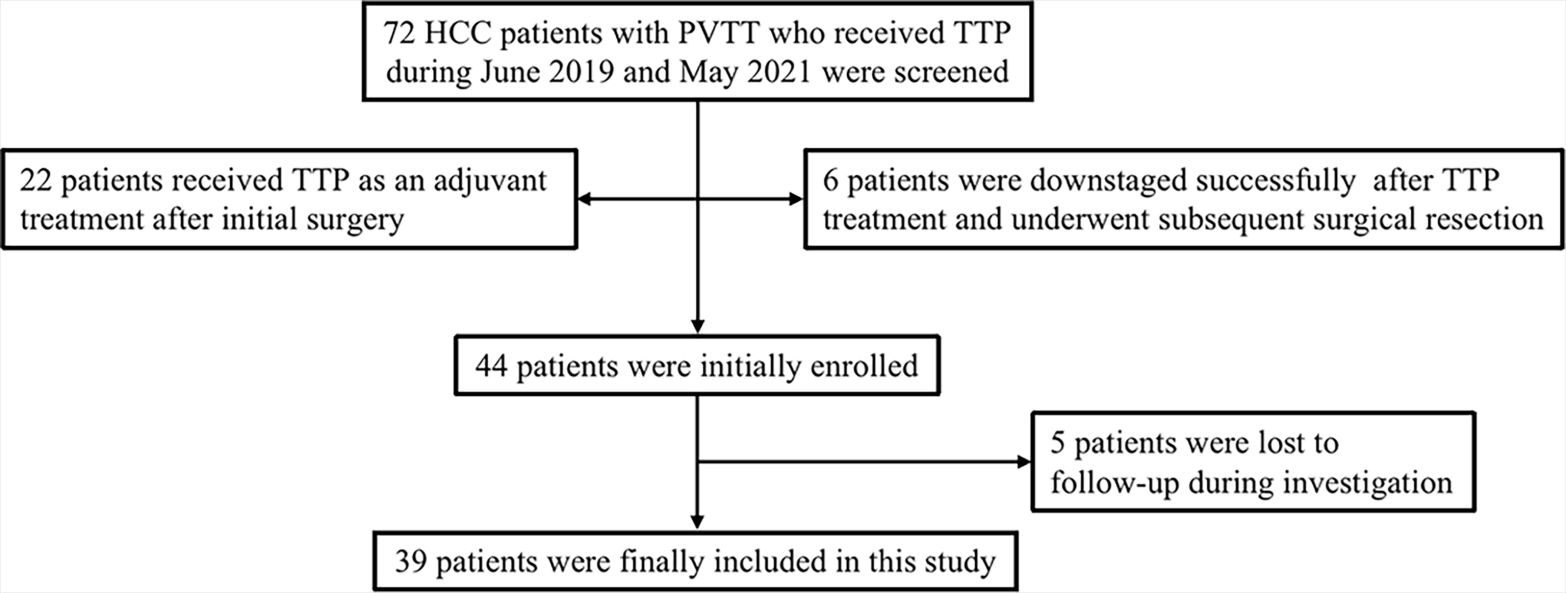
Flow diagram of patient enrollment. HCC, hepatocellular carcinoma; PVTT, portal vein tumor thrombus; TTP, TACE plus antiangiogenic-targeted treatment and anti-PD-1 inhibitors.

From June 2019 to May 2021, 72 advanced HCC patients with PVTT who underwent TTP (TACE + antiangiogenic-targeted therapy + anti-PD-1 antibodies) were identified from the electronic medical system of our hospital. A total of 22 patients received TTP as an adjuvant treatment after surgery. Six patients were treated with TTP as a conversion therapy for subsequent surgical resection. Five patients were lost to follow-up during investigation. Finally, a total of 39 patients who met the eligibility criteria were included in the study. The patients’ identification process is shown in **Figure 1**.

The baseline demographic and clinical characteristics of unresectable HCC patients with PVTT are listed in **Table 1**. The majority of patients had HBV-associated HCC, and 25.6% of patients had Child–Pugh class B liver function. Liver cirrhosis was observed in 89.7% of patients. All patients were classified as BCLC stage C due to major vascular invasion. The median size of the baseline target lesions was 10.0 cm [interquartile range (IQR), 7.5–12.0 cm]. Two-thirds of patients had multiple tumors. With regard to the extent of PVTT, 66.7% and 25.6% of patients had type II and III PVTT, respectively. A total of 10 patients had combined hepatic vein or inferior vena cava tumor thrombus. Regional or distant lymph node metastasis occurred in nearly half of patients. A total of eight patients had extrahepatic disease spread, including five in the lungs and three in the adrenal gland. The median level of α-fetoprotein (AFP) was 1,509 ng/ml (IQR, 10.1–32,280 ng/ml). The median concentration of protein induced by vitamin K absence or antagonist-II (PIVKAII) was 5,413 mAU/ml (IQR, 617–47,033 mAU/ml). Twenty-two patients had an HBV-DNA level exceeding 1,000 copies/ml.

**Table 1:** Baseline demographic and clinical characteristics of unresectable hepatocellular carcinoma (HCC) patients with portal vein tumor thrombus (PVTT).

Characteristics	All patients (n=39)
Age, median (range), years	56 (31–69)
<60	29 (74.4%)
≥60	10 (25.6%)
Gender	
Male	33 (84.6%)
Female	6 (15.4%)
Hypertension	
Yes	7 (17.9%)
No	32 (82.1%)
Diabetes mellitus	
Yes	4 (10.3%)
No	35 (89.7%)
Antiviral therapy	
Yes	22 (56.4%)
No	17 (43.6%)
Child–Pugh class	
A	29 (74.4%)
B	10 (25.6%)
ALBI score, median (Q1, Q3)	-2.14 (-2.47, -1.94)
ALBI grade	
1	6 (15.4%)
2	31 (79.5%)
3	2 (5.1%)
Etiology	
Hepatitis B	38 (97.4%)
Hepatitis C	1 (2.6%)
Cirrhosis	
Yes	35 (89.7%)
No	4 (10.3%)
Tumor number	
Single	13 (33.3%)
Multiple	26 (66.7%)
Tumor size (cm), median (Q1, Q3)	10.0 (7.5–12.0)
≤5	2 (5.1%)
5–10	19 (48.7%)
>10	18 (46.2%)
Tumor distribution	
Left lobe	5 (12.8%)
Right lobe	25 (64.1%)
Bi-lobe	9 (23.1%)
PVTT type	
I	3 (7.7%)
II	26 (66.7%)
III	10 (25.6%)
Combined HVTT/IVCTT, yes	10 (25.6%)
HVTT	8 (20.5%)
IVCTT	2 (5.1%)
Lymph node metastasis	
Yes	19 (48.7%)
No	20 (51.3%)
Extrahepatic spread, yes	8 (20.5%)
Lung	5 (12.8%)
Adrenal gland	3 (7.7%)
Esophagogastric varices	
Presence	19 (48.7%)
Absence	20 (51.3%)
PT (s), median (Q1, Q3)	12.2 (11.9–13.2)
INR, median (Q1, Q3)	1.02 (0.99–1.10)
WBC (*10^9/L), mean ± SD	5.4 ± 2.2
RBC (*10^12/L), mean ± SD	4.3 ± 0.7
HGB (g/L), mean ± SD	131.1 ± 22.1
PLT (g/L), mean ± SD	146.8 ± 70.4
TBil (μmol/L), median (Q1, Q3)	17.3 (13.1–22.6)
ALB (g/L), mean ± SD	38.2 ± 3.8
ALT (U/L), median (Q1, Q3)	41 (29–59)
AST (U/L), median (Q1, Q3)	59 (46–124)
GGT (U/L), median (Q1, Q3)	192 (116–371)
ALP (U/L), median (Q1, Q3)	144 (109–200)
BUN (mmol/L), median (Q1, Q3)	4.52 (3.77–5.02)
Creatinine (μmol/L), median (Q1, Q3)	67 (57–78)
Glucose (mmol/L), median (Q1, Q3)	4.87 (4.37–5.19)
AFP (ng/ml), median (Q1, Q3)	1,509 (10.1–32,280)
<400	15 (38.5%)
≥400	24 (61.5%)
PIVKAII (mAU/ml), median (Q1, Q3)	5,413 (617–47,033)
< 2,050	14 (35.9%)
≥ 2,050	25 (64.1%)
HBV-DNA, copies/ml	
<50	9 (23.7%)
50–1,000	7 (18.4%)
≥1,000	22 (57.9%)

HCC, hepatocellular carcinoma; PVTT, portal vein tumor thrombus; ALBI, albumin-bilirubin; HVTT, hepatic vein tumor thrombus; IVCTT, inferior vena cava tumor thrombus; PT, prothrombin time; INR, international normalized ratio; WBC, white blood cell; RBC, red blood cell; HGB, hemoglobin; PLT, platelet; TBil, total bilirubin; ALB, albumin; ALT, alanine aminotransferase; AST, aspartate aminotransferase; GGT, γ-glutamyltranspeptidase; ALP, alkaline phosphatase; BUN, blood urea nitrogen; AFP, alpha-fetoprotein; PIVKAII, protein induced by vitamin K absence or antagonist-II.

### Number of transarterial chemoembolization procedures and transarterial chemoembolization interval time

A total of 13 patients underwent one session of TACE, and the other 26 patients received two or more sessions of TACE. The median interval between each TACE treatment was 79.8 days (**Table 2**).

**Table 2:** Number of transarterial chemoembolization (TACE) procedures and treatment intervals.

	All patients (n=39)
Number of TACE procedures, n (%)	
1	13 (33.3%)
2	15 (38.5%)
3	8 (20.5%)
4	1 (2.6%)
5	2 (5.1%)
Median interval between TACE (days), mean (SD)	79.8 (53.5)

TACE, transarterial chemoembolization.

### Types of antiangiogenic-targeted drugs and anti-PD-1 antibodies

For antiangiogenic-targeted drugs, 30 patients initially used lenvatinib, and the remaining 9 patients initially used sorafenib. During the treatment course, because of unacceptable AEs or progressed disease, five patients initially using sorafenib converted to lenvatinib or anlotinib, whereas two patients initially using lenvatinib converted to anlotinib or apatinib. As for anti-PD-1 antibodies, 16 used sintilimab, 14 had camrelizumab, 6 were treated with toripalimab, and the other 3 patients received tislelizumab. Among them, one patient who was initially treated with sintilimab converted to camrelizumab due to infusion-related reactions (**Table 3**).

**Table 3:** Types of antiangiogenic-targeted drugs and anti-PD-1 antibodies.

	All patients (n=39)
***Initial treatments***	
Antiangiogenic-targeted drugs	
Sorafenib	9 (23.1%)
Lenvatinib	30 (76.9%)
**Anti-PD-1 antibodies**	
Sintilimab	16 (41.0%)
Toripalimab	6 (15.4%)
Camrelizumab	14 (35.9%)
Tislelizumab	3 (7.7%)
***Whole clinical treatment pathway***	
Antiangiogenic-targeted drugs	
Sorafenib	9 (23.1%)
Lenvatinib	35 (89.7%)
Anlotinib	2 (5.1%)
Apatinib	1 (2.6%)
**Anti-PD-1 antibodies**	
Sintilimab	16 (41.0%)
Toripalimab	6 (15.4%)
Camrelizumab	15 (38.5%)
Tislelizumab	3 (7.7%)

### Change of tumor marker expression

The changes of tumor marker levels from the baseline to the first follow-up after the triple treatment approach are shown in **Table 4**. The median AFP level at the baseline was 1,509 ng/ml, while this level dropped dramatically to 135 ng/ml at the first follow-up (*P* = 0.126). From a dichotomous point, 15 (38.5%) patients had an AFP level not greater than 400 ng/ml at the baseline, whereas 25 (64.1%) patients had an AFP level within 400 ng/ml at the first follow-up after the triple therapy (*P* = 0.023). For PIVKAII, the median baseline level was 5,413 mAU/ml, and this level decreased markedly to 950 mAU/ml at the first follow-up (*P* = 0.003). For HBV-DNA, the median concentration at the baseline was 6,240 copies/ml, which reduced remarkably to 36 copies/ml at the first follow-up (*P* < 0.001).

**Table 4:** Change of tumor marker expression.

	Baseline	First follow-up after TTP treatment	*P-*value
AFP (ng/ml), median (Q1, Q3)	1,509 (10.1–32,280)	135 (4.7–9,427)	0.126
PIVKAII (mAU/ml), median (Q1, Q3)	5,413 (617–47,033)	950 (34–11,386)	**0.003**
HBV-DNA (copies/ml)	6,240 (54.7–532,750)	36 (2–191.3)	**< 0.001**
HCV-RNA (copies/ml)	55,200	150,000	NA
AFP (ng/ml)			**0.023**
<400	15 (38.5%)	25 (64.1%)	
≥400	24 (61.5%)	14 (35.9%)	
PIVKAII (mAU/ml)			**0.007**
<2,050	14 (35.9%)	26 (66.7%)	
≥2,050	25 (64.1%)	13 (33.3%)	
HBV-DNA (copies/ml)			**< 0.001**
<1,000	16 (42.1%)	34 (89.5%)	
≥1,000	22 (57.9%)	4 (10.5%)	

TTP, TACE + antiangiogenic-targeted therapy + anti-PD-1 antibody treatment; AFP, alpha-fetoprotein; PIVKAII, protein induced by vitamin K absence or antagonist-II; HBV-DNA, hepatitis B virus deoxyribonucleic acid; NA, data not available.

P-values in bold denote statistical significance.

### Change of liver function

As shown in **Table 5**, the Child–Pugh class and the ALBI grade were used to assess the hepatic functional reserve in the baseline and the first follow-up after the triple therapy. There was no significant change of liver function between the baseline and the first follow-up after treatment in either the Child–Pugh class or the ALBI grade (*P* = 0.151 and *P* = 0.842).

**Table 5:** Change of liver function.

	Baseline	First follow-up after TTP treatment	*P-*value
Child–Pugh class			0.151
A	29 (74.4%)	34 (87.2%)	
B	10 (25.6%)	5 (12.8%)	
ALBI grade			0.842
1	6 (15.4%)	7 (17.9%)	
2	31 (79.5%)	29 (74.4%)	
3	2 (5.1%)	3 (7.7%)	

TTP, TACE + antiangiogenic targeted therapy + anti-PD-1 antibody treatment; ALBI, albumin–bilirubin.

### Additional treatments aside from the triple therapy

As shown in **Table 6**, five (12.8%) patients received additional radiation treatment that was targeted at liver lesions or extrahepatic metastasis. Six (15.4%) patients underwent synchronous percutaneous microwave coagulation therapy. One patient ceased the triple therapy after six cycles of PD-1 treatment and converted to atezolizumab plus bevacizumab (T+A therapy). Four (10.3%) patients received the best supportive care during the treatment course due to liver function deterioration.

**Table 6:** Additional treatments aside from TACE + antiangiogenic targeted therapy + anti-PD-1 antibody treatment.

	All patients (n=39)
Radiotherapy	5 (12.8%)
PMCT	6 (15.4%)
T+A	1 (2.6%)
BSC	4 (10.3%)

TTP, TACE + antiangiogenic targeted therapy + anti-PD-1 antibody treatment; PMCT, percutaneous microwave coagulation therapy; T+A, atezolizumab combined with bevacizumab; BSC, best supportive care.

### Efficacy outcomes

**Figure 2:**
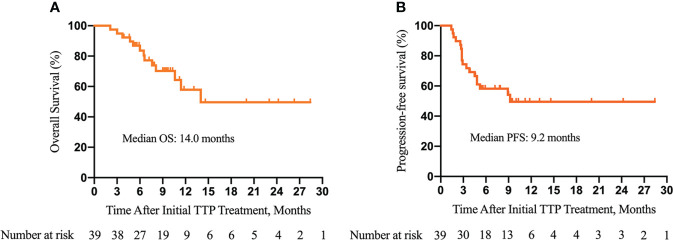
Kaplan–Meier survival curves of overall survival (OS) **(A)** and progression-free survival (PFS) **(B)** of patients with unresectable hepatocellular carcinoma and portal vein tumor thrombus (PVTT) who underwent the triple therapy. TTP, transarterial chemoembolization (TACE) plus antiangiogenic-targeted treatment and anti-PD-1 inhibitors.

As of the data cutoff on 30 August 2021, the median follow-up time was 10.0 (3.9–28.4) months. Disease progression occurred in 18 (46.2%) patients, and 13 (33.3%) patients died. The median OS was 14.0 months. The 3-, 6-, and 12-month OS was 94.9%, 83.7%, and 57.9%, respectively (**Figure 2A**). The median PFS was 9.2 months. The 3-, 6-, and 12-month PFS was 74.4%, 58.2%, and 49.6%, respectively (**Figure 2B**). The best tumor response is summarized in **Table 7**. The ORR and DCR were 35.9% and 74.4% according to the RECIST 1.1 and 48.7% and 84.6% according to mRECIST criteria, respectively.

**Table 7:** Summary of the best tumor response.

	All patients (n=39) mRECIST	All patients (n=39) RECIST 1.1
CR	3 (7.7%)	3 (7.7%)
PR	16 (41.0%)	11 (28.2%)
SD	14 (35.9%)	15 (38.5%)
PD	6 (15.4%)	10 (25.6%)
ORR	19 (48.7%)	14 (35.9%)
DCR	33 (84.6%)	29 (74.4%)

Tumor response was assessed using mRECIST and RECIST 1.1 criteria, respectively.

Data are presented as n (%).

CR, complete response; PR, partial response; SD, stable disease; PD, progressive disease; ORR, objective response rate; DCR, disease control rate.

**Figure 3:**
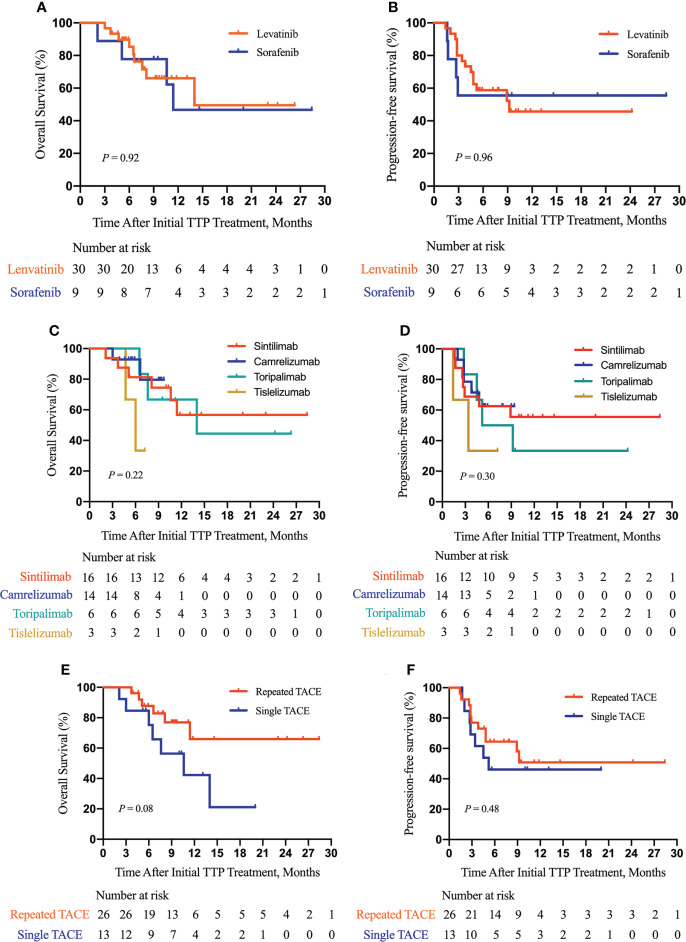
Subgroup survival analysis according to the type of molecular targeted drugs, the type of PD-1 inhibitors, and the number of TACE procedures. Subgroup analysis of OS **(A)** and PFS **(B)** according to the type of molecular targeted drugs; a subgroup analysis of OS **(C)** and PFS **(D)** according to the type of PD-1 inhibitors; a subgroup analysis of OS **(E)** and PFS **(F)** according to the number of TACE procedures. TTP, TACE plus antiangiogenic-targeted treatment and anti-PD-1 inhibitors; TACE, transarterial chemoembolization.

**Figure 4:**
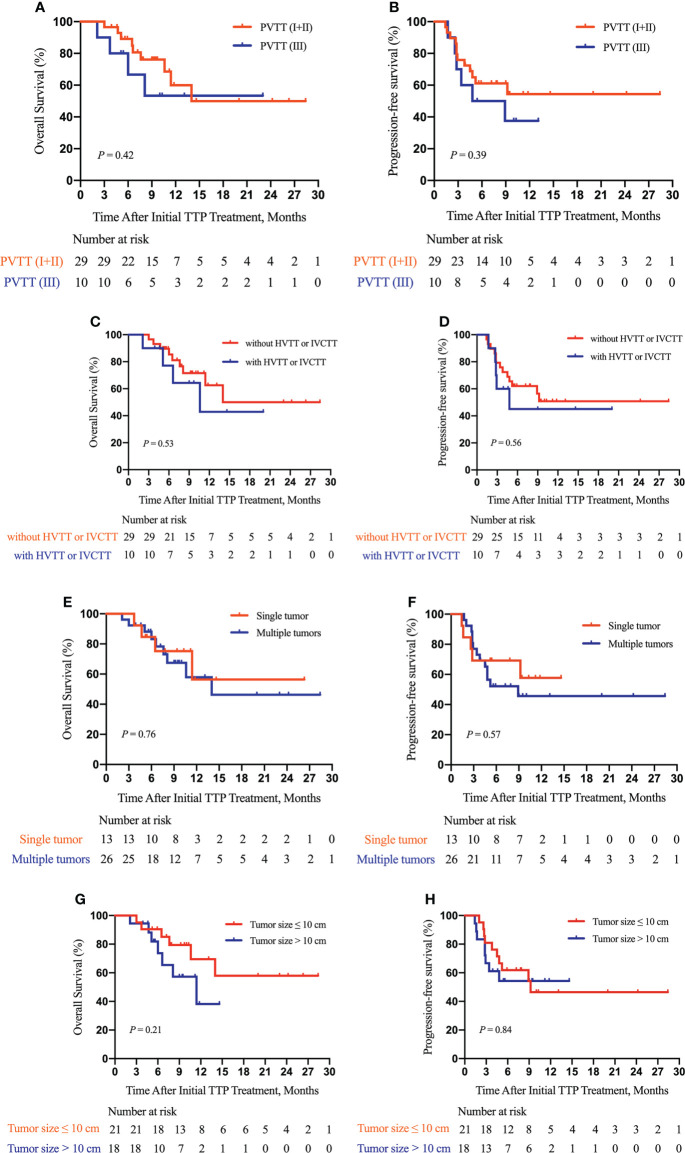
Subgroup survival analysis according to the type of PVTT, the presence or absence of hepatic vein tumor thrombus (HVTT)/inferior vena cava tumor thrombus (IVCTT), the tumor number, and tumor size. Subgroup analysis of OS **(A)** and PFS **(B)** according to the type of PVTT; a subgroup analysis of OS **(C)** and PFS **(D)** according to the presence or absence of HVTT/IVCTT; a subgroup analysis of OS **(E)** and PFS **(F)** according to the tumor number; a subgroup analysis of OS **(G)** and PFS **(H)** according to tumor size. TTP, TACE plus anti-angiogenic-targeted treatment and anti-PD-1 inhibitors; PVTT, portal vein tumor thrombus; HVTT, hepatic vein tumor thrombus; IVCTT, inferior vena cava tumor thrombus.

Furthermore, as shown in **Figure 3**, we did subgroup survival analysis according to administered drugs and the number of TACE procedures. No significant differences in OS and PFS were observed for patients initially using sorafenib or lenvatinib (*P* = 0.92, **Figure 3A**; *P* = 0.96, **Figure 3B**). Similarly, there were no marked OS and PFS differences among patients who were treated with various anti-PD-1 inhibitors (*P* = 0.22, **Figure 3C**; *P* = 0.30, **Figure 3D**). Repeated TACE appeared to show survival advantage over a single TACE treatment, but the statistical difference was not significant (*P* = 0.08, **Figure 3E**; *P* = 0.48, **Figure 3F**). The results of subgroup survival analysis according to (I) the types of PVTT, (II) the presence or absence of concurrent HVTT/IVCTT, (III) the tumor number, and (IV) tumor size are illustrated in **Figure 4**. As shown in **Figure S1**, patients who received additional treatments (radiotherapy, percutaneous microwave coagulation therapy, or T+A) had significantly better OS than those who did not (*P* = 0.046, **Figure S1A**). The PFS of patients who underwent additional treatments was also better compared with that of patients who did not but without statistical significance (*P* = 0.14, **Figure S1B**). The results of the univariate analysis of OS and PFS are displayed in **Table S1**. The association between the different cycles of TTP therapy and liver function and tumor marker expression change can be seen in **Figure S2**.

### Safety outcomes

Treatment- and laboratory-related AEs including frequency and the severity grade were evaluated according to CTCAE, version 5.0. A total of 34 (87.2%) patients experienced at least one treatment-related AE, and 8 (20.5%) patients experienced grade 3/4 treatment-related AEs. The most common treatment-emergent AE of all grade was hypertension (46.2%) followed by diarrhea (35.9%), fatigue (30.8%), PPE (25.6%), weight loss (23.1%), skin pruritis (20.5%), and nausea (20.5%). The most common laboratory-related AE of all grades was decreased albumin (53.8%) followed by thrombocytopenia (41.0%), increased aspartate aminotransferase (30.8%), increased γ-glutamyltranspeptidase (25.6%), increased alanine aminotransferase (23.1%), neutropenia (23.1%), and hyperbilirubinemia (20.5%). In addition, the most common grade 3/4 treatment-emergent AE was hypertension (20.5%), while the most common grade 3/4 laboratory-related AE was thrombocytopenia (10.3%) (**Table 8**).

**Table 8:** Summary of adverse events.

Adverse events	Any Gradesn (%)	Grade 3/4n (%)
**Treatment-related AEs**		
**Skin and subcutaneous tissue**		
Palmar-plantar erythrodysesthesia	10 (25.6%)	0 (0.0%)
Skin pruritus	8 (20.5%)	1 (2.6%)
Skin rash	6 (15.4%)	1 (2.6%)
**Cardiovascular system**		
Hypertension	18 (46.2%)	8 (20.5%)
**Digestive system**		
Diarrhea	14 (35.9%)	1 (2.6%)
Nausea	8 (20.5%)	1 (2.6%)
Gastrointestinal hemorrhage	1 (2.6%)	0 (0.0%)
**Kidney and urinary system**		
Proteinuria	3 (7.7%)	2 (5.1%)
**Nervous system**		
Headache	4 (10.3%)	0 (0.0%)
**Chest and mediastinum**		
Dysphonia	6 (15.4%)	0 (0.0%)
**Endocrine system**		
Hypothyroidism	4 (10.3%)	0 (0.0%)
**Metabolism and nutrition**		
Decreased appetite	7 (17.9%)	1 (2.6%)
Weight loss	9 (23.1%)	0 (0.0%)
**Systemic symptoms**		
Fatigue	12 (30.8%)	0 (0.0%)
Fever	4 (10.3%)	1 (2.6%)
Peripheral edema	2 (5.1%)	0 (0.0%)
**Laboratory-related AEs**		
**Blood biochemistry**		
Hyperbilirubinemia	8 (20.5%)	1 (2.6%)
Alanine aminotransferase increased	9 (23.1%)	1 (2.6%)
Aspartate aminotransferase increased	12 (30.8%)	1 (2.6%)
Albumin decreased	21 (53.8%)	0 (0.0%)
γ-Glutamyltranspeptidase increased	10 (25.6%)	1 (2.6%)
Creatinine increased	1 (2.6%)	0 (0.0%)
**Blood routine tests**		
Anemia	7 (17.9%)	0 (0.0%)
Thrombocytopenia	16 (41.0%)	4 (10.3%)
Neutropenia	9 (23.1%)	0 (0.0%)

The adverse events related with antiangiogenic-targeted drugs and anti-PD-1 antibodies were graded according to Common Terminology Criteria Adverse Events (CTCAE) Version 5.0.

## Discussion

PVTT remains as the bottleneck in the treatment of HCC, which contributes to high recurrence rates and a poor prognosis. According to the BCLC staging system, HCC with PVTT is graded as the advanced stage, which often precludes the opportunity of surgical resection (6). As a chance for cure, molecular-targeted therapy and PD-1 blockades have revolutionized cancer treatment and drastically changed the treatment landscape for advanced HCC with PVTT.

The gradually evolving role of systemic therapy for advanced HCC has been documented (16, 17). The regulating effect of small molecular-targeted drugs on the tumor microenvironment tends to increase the therapeutic effect of PD-1 inhibitors and vice versa (18). This triggers the combination therapy of immune checkpoint inhibitors (ICIs) with angiogenic targeted drugs for unresectable HCC. A meta-analysis showed that ORR and DCR were 29% (95% CI 0.15–0.43) and 77% (95% CI 0.70–0.84) for patients treated with PD-1/PD-L1 monoclonal antibodies combined with anti-VEGF agents (19). A single-arm retrospective study showed that sintilimab plus tyrosine kinase inhibitors (TKIs) exhibited promising efficacy with tolerable adverse reactions in unresectable HCC (20).

It is notable that locoregional therapies (LRTs) can induce the release of inflammatory and proangiogenic factors and neoantigens and increase the expression of PD-1 and PD-L1; systemic drugs are administered as an adjuvant therapy in combination with LRTs (21–23). Two prospective studies showed that TACE plus lenvatinib is safe, is well tolerated, and has satisfactory efficacy for the treatment of HCC with PVTT (24, 25). Stereotactic body radiotherapy (SBRT) combined with ICIs was reported to have an impressive tumor control capability for patients with unresectable HCC of large tumors (26). In addition, propensity score matching (PSM) analysis suggested that bigeminal therapy with PD-1 blockade plus radiofrequency ablation (RFA) was superior to RFA alone for the long-term survival of recurrent HCC patients (27).

Recently, a triple therapy of TKIs in combination with PD-1 inhibitors and LRTs to treat unresectable HCC patients has gathered much attention and yielded substantial clinical benefits (28, 29). Dai et al. (30) found that the median OS and PFS were 13.0 and 5.0 months, respectively, for inoperable HCC patients who underwent sintilimab combined with sorafenib and TACE. The ORR and DCR were 28.6% and 80.0%, respectively. Teng et al. (31) reported the therapeutic efficacy of TACE plus ICIs and lenvatinib in unresectable HCC. The results showed that the ORR and DCR were 54.9% and 84.3%, respectively, and the median PFS was 8.5 months. Nearly one-third of the patients experienced grade ≥ 3 AEs. Cai et al. (32) showed that the TACE-lenvatinib-PD-1 inhibitor (TACE-L-P) group had a median OS of 16.9 months and a median PFS of 7.3 months. The ORR and DCR of the TACE-L-P group were 56.1% and 85.4%, respectively. A multicenter study recorded that the investigator and blinded independent central review–assessed ORR were 80.6% and 77.4%, respectively, for unresectable HCC patients who underwent the triple therapy. Of 62 included cases, 33 (53.2%) patients reached the standard of successful conversion to resectable disease; 16 (25.8%) and 24 (38.7%) patients had complete and major pathological response, respectively (33).

To the best of our knowledge, our study is the first to explore the clinical efficacy and safety of the triple therapeutic approach consisting of TACE, anti-angiogenic therapy, and PD-1 inhibitors in unresectable HCC patients with PVTT. Regarding the safety of the triple therapy, our study showed that 34 (87.2%) patients experienced at least one treatment-related AE, 8 (20.5%) patients experienced grade 3/4 treatment-related AEs, and no treatment-related death occurred. The incidence rates of overall and severe AEs were similar to the previous reports, which proved the acceptable safety profile of the triple therapy in this patient population. With respect to the treatment response and survival outcomes following the triple therapy, the ORR and DCR based on the mRECIST criteria were 48.7% and 84.6%, respectively; and the median OS and PFS were 14.0 and 9.2 months, respectively. The efficacy outcomes were comparable to some previous results (13, 30–32). However, our tumor response rates were lower than those reported by Wu et al. (33). The patient selection difference may be the possible reason as 56.5% patients in their study cohort did not have PVTT. Therefore, based on the above analysis, the triple therapy could result in good efficacy for unresectable HCC with PVTT. TACE, molecular-targeted drugs, and ICIs may have synergistic effects and augment the antitumor activity mutually. However, the mechanisms underlying the triple therapy still need further investigations.

The present study had several limitations. First, this was a retrospective study with a limited sample size and a short follow-up time, contributing to potential selection bias and relatively insufficient medical evidence. Second, this study was a single-arm study with no control group, so it was impossible to compare the efficacy and safety of this triple therapy with other combined therapeutic approaches. Third, the substantial heterogeneity of the study population and the inconformity of treatment regimens may influence the interpretation of our findings. Thus, prospective studies with a large sample size are required to determine whether combining TACE, antiangiogenic agents, and PD-1 inhibitors potentiates clinical efficacy.

## Conclusion

TACE in combination with antiangiogenic-targeted therapy and PD-1-targeted immunotherapy displayed promising tumor control rates with well-tolerated toxicity in unresectable HCC patients associated with PVTT in the real world. Thus, this triple therapeutic strategy may be an ideal treatment option for these patients. In the future, the identification of molecular biomarkers to select patients who are most likely to benefit from the triple therapy should be highlighted.

## Data availability statement

The raw data supporting the conclusions of this article will be made available by the authors, without undue reservation.

## Ethics statement

The studies involving human participants were reviewed and approved by institutional ethics committee of Eastern Hepatobiliary Surgery Hospital. The ethics committee waived the requirement of written informed consent for participation.

## Author contributions

Conceptualization and design: S-QC, JZ, J-KF, Z-HL, Z-GF. Administrative support and funding acquisition: S-QC. Provision of study materials or patients: S-QC, JZ, Z-GF, Z-TC, J-XS, KW, Y-QC, L-PZ, JS, W-XG. Collection and assembly of data: J-KF, Z-HL, Z-GF, H-FZ, Y-JX. Data analysis and interpretation: J-KF, Z-HL, Z-GF. Manuscript writing: J-KF. Manuscript review and editing: S-QC, JZ. All authors contributed to the article and approved the submitted version.

## Funding

This work was supported by the Clinical Research Plan of Shanghai Hospital Development Center (No. SHDC2020CR1004A), the Key Project of the National Natural Science Foundation of China (No: 81730097), the National Natural Science Foundation of China (No: 82072618), and the National Key Research and Development Program of China (2022YFC2503700; 2022YFC2503701; 2022YFC2503703; 2022YFC2503705).

## Conflict of interest

The authors declare that the research was conducted in the absence of any commercial or financial relationships that could be construed as a potential conflict of interest.

## Publisher’s note

All claims expressed in this article are solely those of the authors and do not necessarily represent those of their affiliated organizations, or those of the publisher, the editors and the reviewers. Any product that may be evaluated in this article, or claim that may be made by its manufacturer, is not guaranteed or endorsed by the publisher.
