# Supplementary figures and images for: Efficacy and safety of transarterial chemoembolization plus antiangiogenic- targeted therapy and immune checkpoint inhibitors for unresectable hepatocellular carcinoma with portal vein tumor thrombus in the real world
Source: Front Oncol. 2022 Nov 25;12:954203. doi: 10.3389/fonc.2022.954203 (PMC9732723; doi:10.3389/fonc.2022.954203)

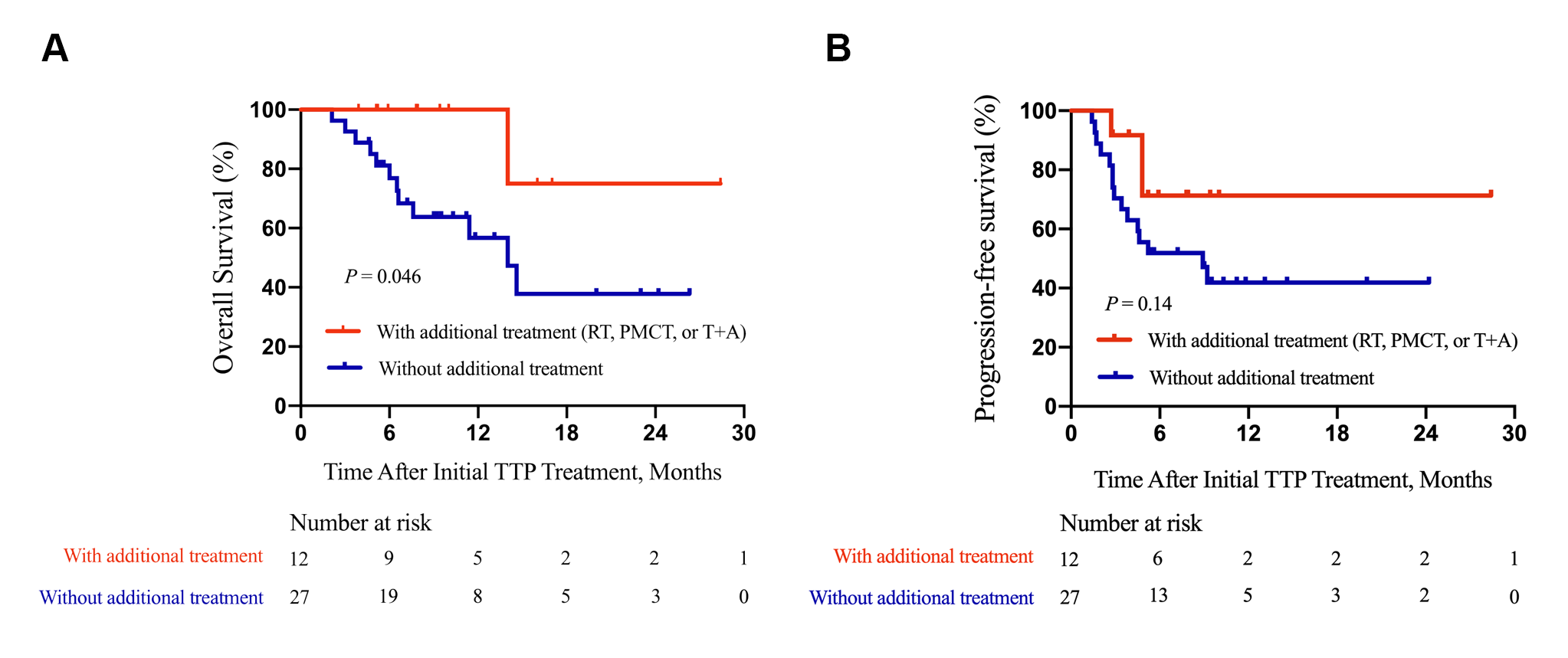

Supplement: Supplementary file 1 [file Image_1.tif]

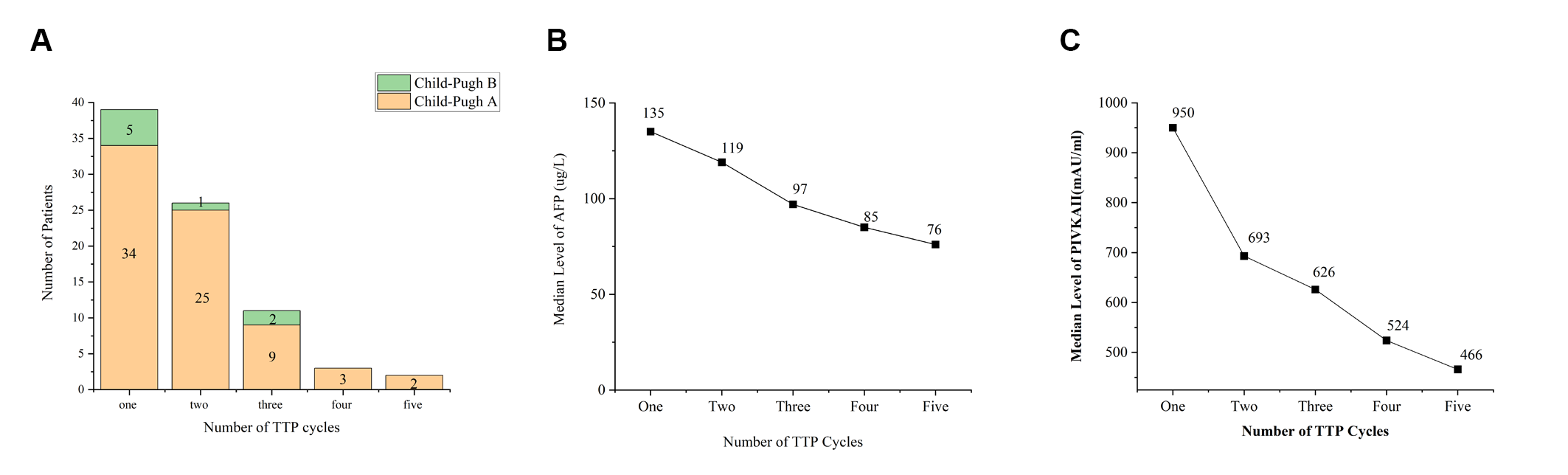

Supplement: Supplementary file 2 [file Image_2.tif]
