# Supplementary material for: Efficacy and safety of transarterial chemoembolization plus antiangiogenic- targeted therapy and immune checkpoint inhibitors for unresectable hepatocellular carcinoma with portal vein tumor thrombus in the real world
Source: Front Oncol. 2022 Nov 25;12:954203. doi: 10.3389/fonc.2022.954203 (PMC9732723; doi:10.3389/fonc.2022.954203)
Supplement: Supplementary file 3 [file Table_1.docx]

| **Variables** | Overall survival | | Progression-free survival | |
| --- | --- | --- | --- | --- |
|  | Univariate analysis | | Univariate analysis | |
|  | HR (95% CI) | *P* | HR (95% CI) | *P* |
| Tumor number  (multiple vs. single) | 1.197 (0.368–3.897) | 0.765 | 1.362 (0.482–3.844) | 0.560 |
| Tumor size  (> 10 vs. ≤ 10 cm) | 2.019 (0.664–6.139) | 0.216 | 1.106 (0.436–2.806) | 0.832 |
| AFP  (≥ 400 vs. < 400 ng/mL) | 2.423 (0.662–8.869) | 0.181 | 1.501 (0.563–4.003) | 0.417 |
| PIVKAII  (≥ 2050 vs. < 2050 mAU/mL) | 3.480 (0.769–15.747) | 0.105 | 1.863 (0.662–5.239) | 0.239 |
| HBV-DNA  (≥ 1000 vs. < 1000 copies/mL) | 1.717 (0.526–5.598) | 0.370 | 2.205 (0.784–6.200) | 0.134 |
| PVTT type  (III vs. I–II) | 1.621 (0.497–5.288) | 0.424 | 1.544 (0.579–4.119) | 0.385 |
| Child-Pugh class  (B vs. A) | 1.897 (0.619–5.809) | 0.262 | 1.609 (0.603–4.291) | 0.342 |

Supplementary Table 1. Univariate analysis for overall survival and progression-free survival

**Abbreviations:** AFP, alpha-fetoprotein; PIVKAII, protein induced by vitamin K absence or antagonist-II; PVTT, portal vein tumor thrombus.
